# Comprehensive immunoprofile analysis of prognostic markers in pancreaticobiliary tract cancers

**DOI:** 10.1002/cam4.5530

**Published:** 2023-01-17

**Authors:** Ji Eun Kim, Hyemin Kim, Binnari Kim, Hye Gyo Chung, Hwe Hoon Chung, Kyoung Mee Kim, Seong Hyun Kim, Woo Kyoung Jeong, Young Kon Kim, Ji Hye Min, Jin Seok Heo, In Woong Han, Sang Hyun Shin, Hee Chul Park, Jeong Il Yu, Joon Oh Park, Seung Tae Kim, Jung Yong Hong, Se‐Hoon Lee, Kwang Hyuck Lee, Jong Kyun Lee, Kyu Taek Lee, Kee‐Taek Jang, Joo Kyung Park

**Affiliations:** ^1^ Department of Medicine, Samsung Medical Center Sungkyunkwan University School of Medicine Seoul South Korea; ^2^ Medical Research Institute Sungkyunkwan University School of Medicine Seoul South Korea; ^3^ Department of Pathology, Ulsan University Hospital University of Ulsan College of Medicine Ulsan South Korea; ^4^ Department of Pathology, Samsung Medical Center Sungkyunkwan University School of Medicine Seoul South Korea; ^5^ Department of Radiology, Samsung Medical Center Sungkyunkwan University School of Medicine Seoul South Korea; ^6^ Department of Hepato Biliary Pancreatic Surgery, Samsung Medical Center Sungkyunkwan University School of Medicine Seoul South Korea; ^7^ Department of Radiation Oncology, Samsung Medical Center Sungkyunkwan University School of Medicine Seoul South Korea; ^8^ Department of Hematology/Oncology, Samsung Medical Center Sungkyunkwan University School of Medicine Seoul South Korea; ^9^ Department of Health Sciences and Technology, SAIHST Sungkyunkwan University Seoul South Korea

**Keywords:** CD8, CXCL13, FOXP3, pancreaticobiliary tract cancer, PD‐1, PD‐L1, prognosis

## Abstract

Pancreaticobiliary tract cancer has a poor prognosis with unmet needs in a new target treatment. Some studies have reported that an enhancement of T‐cell immunity is associated with a good prognosis. The aim of this study is to investigate the immunoprofile as a prognostic marker of pancreaticobiliary tract cancers. Unresectable pancreatic ductal adenocarcinoma (PDAC, *n* = 80) and biliary tract cancer (BTC, *n* = 74) diagnosed between January 2012 and December 2018 in Samsung Medical Center were analyzed. Expression levels of CD8, FOXP3, PD‐1, PD‐L1, and CXCL13 in PDAC and BTC tissue samples were examined with immunohistochemical staining, which was evaluated with various clinical factors. In PDAC, higher degree of PD‐L1 expression was significantly associated with shorter overall survival (OS) (*p* = 0.0095). On the other hand, higher infiltrations of PD‐1^+^ immune cells (*p* = 0.0002) and CD8^+^ T cells (*p* = 0.0067) were associated with longer OS. In BTC, higher FOXP3^+^ (*p* = 0.0343) and CD8^+^ (*p* = 0.0028) cell infiltrations were associated with better survival. Low infiltration of CD8^+^ (*p* = 0.0148), FOXP3^+^ (*p* = 0.0208), PD‐1^+^ (*p* = 0.0318) cells in PDAC, and FOXP3^+^ cells (*p* = 0.005) in BTC were considerably related to metastasis. In a combined evaluation of clinical factors and immunoprofiles, univariate analysis revealed that operation after chemotherapy (*p* < 0.0001), mass size (*p* = 0.0004), metastasis (*p* = 0.006), PD‐L1 (*p* < 0.0001), PD‐1 (*p* = 0.003) and CD8 (*p* = 0.0063) was significantly associated with OS in PDAC, and CD8 (*p* = 0.007) was statistically related to OS in BTC. In multivariate analysis, prognostic factors were operation after chemotherapy (*p* = 0.021) in PDAC and CD8 (*p* = 0.037) in BTC. Therefore, immunoprofile analysis of cells expressing CD8, FOXP3, PD‐1, and PD‐L1 might have prognostic values in patients with pancreaticobiliary tract cancers.

## Graphical abstract

Prognostic immunoprofile analysis of CD8, FOXP3, PD‐1, and PD‐L1 in pancreaticobiliary tract cancer.

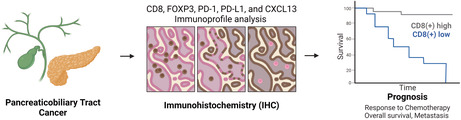

## 1 INTRODUCTION

Pancreaticobiliary tract cancer has a poor prognosis with delayed detection, and there is an unmet need for a new target treatment and a notable prognostic marker to sustainably improve the survival.^1^ Since the Food and Drug Administration (FDA)'s approval of the isocitrate dehydrogenase (IDH) 1 inhibitor ivosidenib in August 2021, there has been increasing interest in targeted therapy for biliary tract cancer patients harboring fibroblast growth factor receptor (FGFR) 2 fusions, neurotrophic receptor tyrosine kinase (NTRK) fusions, B‐raf kinase (BRAF) V600E mutations, and human epidermal growth factor‐2 (HER‐2) amplifications.^2^, ^3^ At the same time, immunotherapy with immune checkpoint inhibitors is also being used in relapsed cholangiocarcinoma. Olaparib, a Poly (ADP‐ribose) polymerase (PARP) inhibitor, was approved by FDA as first‐line targeted therapy for metastatic pancreatic cancer patients with germline breast and ovarian cancer syndrome (BRCA) mutation.^4^ A molecular targeting treatment or immunotherapy paradigm should be an appropriate treatment plan, but it is still insufficient to prove it. Cancer cells can secrete anti‐inflammatory cytokines and antiangiogenic cytokines to create an immune suppressive tumor microenvironment (TME), which promote the formation, progression, and metastasis of cancer.^5^ Various T‐cell subtypes, tertiary lymphoid structures, and cytokines like interferon‐γ are involved in the immunologic reaction of TME.^6^ If actions by several cytokines are unregulated, the balance can be broken and immune surveillance can be avoided. Previous studies have reported that rare long‐term survival in pancreaticobiliary cancer may be related to the enhanced T‐cell immunity.^7^

Cytotoxic CD8^+^ T cells are the most powerful effectors in the anticancer immune response.^8^ In addition, during immune evasion, regulatory T cells (Treg) can secrete interleukin‐2, causing apoptosis of responder T cells.^9^ Also, C‐X‐C Motif Chemokine Ligand 13 (CXCL13):C‐X‐C chemokine receptor type 5 (CXCR5) axis orchestrates cell–cell interactions that regulate lymphocyte infiltration within the TME, thereby determining responsiveness to cytotoxic and immune‐targeted therapies.^10^ The positive programmed death‐ligand 1 (PD‐L1) expression accompanied profiles of lymphocyte exhaustion, enriched inhibitory molecules and pro‐tumor populations and down‐modulation of most MHC class I members was correlated with a poor overall survival (OS) outcome in pancreatic cancer patients.^11^, ^12^ PD‐L1 expression was inversely correlated with tumor‐infiltrating T lymphocytes, particularly CD8^+^ T cells in pancreatic cancer.^12^ And PD‐L1 expression in over 5% of cholangiocarcinoma cells was associated with shortened survival.^13^ In addition, FOXP3 was analyzed together with tumor infiltrating lymphocytes (TILs) to investigate the communication mechanism between FOXP3^+^ cells and effector T cells in TME.^14^ CXCL13 is theoretically known as tertiary lymphoid structures which are essential sites for the initiation and/or maintenance of the local and systemic T‐ and B‐cell responses against tumors, and is associated with a favorable clinical outcome for cancer patients. It has not been practically applied to clinical practice, only results in experimental mice.^15^

The aim of this study was to analyze the expression of immune‐related markers in pancreatic ductal adenocarcinoma (PDAC) and biliary tract cancers (BTC) to find prognostic markers for patients with pancreaticobiliary tract cancers.

## 2 MATERIALS AND METHODS

### 2.1 Study patients

This was a retrospective cohort study of patients registered in Samsung Medical Center (SMC), Seoul, South Korea. We first screened total 999 patients diagnosed with unresectable PDAC or BTC between January 2012 and December 2018 and then finally selected 80 PDAC patients and BTC 74 patients. Following variables were collected by reviewing electronic medical records: age, gender, body mass index, Eastern Cooperative oncology Group (ECOG) performance status, carcinoembryonic antigen (CEA), carbohydrate antigen (CA) 19–9, smoking, operation, mass size, and metastasis. The study was approved by the Ethics Committee of SMC (IRB No. 2020‐07‐012) and conducted in accordance with the principles of the Declaration of Helsinki. As this study used only de‐identified data routinely collected during hospital visits, the requirement to obtain informed patient consent was waived. PDAC specimen tissues were obtained through Endoscopic ultrasonography (EUS)‐guided fine needle biopsy (FNB). And BTC specimen tissues was obtained through liver biopsy with intrahepatic metastasis. Tumor volume of specimens were examined with hematoxylin and eosin staining by pathologists.

### 2.2 Immunostaining

For PD‐1 immunohistochemistry (IHC), paraffin‐embedded tumor sections were dewaxed in xylene and ethanol and autoclaved for 24 min in an antigen retrieval solution to retrieve their antigen epitopes. Tissue sections were incubated at room temperature for 24 min in the Ventana BenchMark XT system (Ventana Medical Systems, Roche) with anti‐PD‐1 (clone NAT105; Ventana Medical Systems). The secondary antibody was incubated with a OptiView DAB IHC detection kit (Ventana Medical Systems) for 12 min. For FOXP3 IHC test, deparaffinized tissues were autoclaved for 20 min in an antigen retrieval solution at 97°C and then incubated with anti‐FOXP3 (clone 236A/E7; Abcam) for 15 min in a BOND‐MAX autoimmunostainer (Leica Biosystem). They were then incubated with a secondary antibody for 10 min in BOND‐MAX autoimmunostainer using BOND polymer refine detection kit (Leica Biosystem). For CD8 IHC test, deparaffinized tissues were autoclaved for 60 min in an antigen retrieval solution and then incubated with anti‐CD8 (clone SP57; Ventana Medical Systems, Roche) for 24 min in Ventana BenchMark XT. These tissues were then incubated with a secondary antibody using an ultraview universal DAB detection kit (Ventana Medical Systems, Roche). For CXCL13 IHC test, deparaffinized tissues were autoclaved for 60 min in an antigen retrieval solution and then incubated with anti‐CXCL13 (R&D Systems) for 20 min in a BOND‐MAX autoimmunostainer (Leica Biosystem). These tissues were then incubated with a secondary antibody for 30 min in the BOND‐MAX autoimmunostainer. PD‐L1 IHC (22C3 PharmDx, DAKO) was performed according to the manufacturer's recommendations. Stained tissue slides were digitally scanned using an Aperio ScanScope® AT System (Leica Microsystems). Tonsil tissue was used as a positive control for the markers. Negative control followed the same staining protocol without the addition of a primary antibody. Detailed information of antibodies and detection kits listed in Table S1.

### 2.3 Pathological scoring

Stained slides were evaluated by light microscopy at x200 magnification by a pathologist blinded to patients' clinicopathologic data. For PD‐1, CD8, FOXP3, and CXCL13 staining, we evaluated tumor‐infiltrating lymphocytes in three representative fields containing the largest amount of tumor in biopsy samples, and containing invasive fronts in surgical samples.^7^ The tumor‐infiltrating lymphocytes showing positivity were expressed as mean (cells per field) of the numbers measured in three fields. For CXCL13, we also evaluated expression of tumor cells by multiplying staining intensity, and percentage of positive tumor cells among the total number of tumor cells. The intensity of staining was grade as 0–3 (0, no staining; 1, weak staining; 2, moderate staining; and 3, strong staining). The percentage of positive staining cells was recorded from 0 to 100%. Therefore, the total CXCL13 score was converted to a score of 300 by examining two different parts (intensity and percentage) of the sample. For PD‐L1, we evaluated the percentage of positive tumor cells with partial or complete membranous staining at any intensity, relative to all viable tumor cells.^16^

### 2.4 Statistical analyses

Continuous variables are expressed as mean ± standard deviation or median with ranges. For comparison of continuous variables, Student's *t*‐test or Mann–Whitney test was used. Categorical variables are expressed as counts with percentages. Fisher's exact test was used to compare categorical variables. The Kaplan–Meier curve with log‐rank test was used to compare survival rate. Cox regression was performed to identify factors associated with survival. A *p*‐value of less than 0.05 was regarded as statistically significant. Statistical analyses were performed with SAS version 9.4 (SAS Institute), R 4.0.4 (http://www.R‐project.org/), and GraphPad Prism 8.0 (GraphPad Software Inc.).

## 3 RESULTS

### 3.1 Baseline characteristics of study subjects

Baseline characteristics of study subjects including 80 patients with PDAC and 74 patients with BTC are summarized in Table 1. Mean age was 62 years for PDAC and 64 years for BTC. The proportion of men was higher than that of women in both PDAC (63.7%) and BTC (59.5%). Most were with ECOG 0 at diagnosis, and nobody showed ECOG 3 in either group. Median CEA and CA 19‐9 were 2.56 ng/mL [1.25–5.70] and 153.21 U/mL [17.35–5318.12] in PDAC group and 3.95 ng/mL [1.24–9.49] and 397.34 U/mL [38.97–7105.51] in BTC group, respectively. Hepatic metastasis was found in 45.9% of cases in the PDAC group and 39.2% in the BTC group. The distribution of metastasis in other organs except liver was 15.3% in PDAC and 18.9% in BTC. The proportion of patients with palliative operation or cytoreductive operation was 16.2% in PDAC and 8.1% in BTC. Only one patient was not received a chemotherapy.

**TABLE 1:** Baseline characteristics of subjects in this study

Characteristics	Pancreatic cancer (*n* = 80)	Biliary tract cancer (*n* = 74)
Age		
Mean ± SD	61.61 ± 9.95	63.82 ± 11.31
Sex (%)		
Male	51 (63.7)	44 (59.5)
Female	29 (36.2)	30 (40.5)
BMI		
Mean ± SD	23.16 ± 3.33	23.14 ± 2.82
Smoking		
Non‐smoker	45 (56.2)	37 (50.0)
Ex‐smoker	20 (25.0)	27 (36.5)
Smoker	15 (18.8)	10 (13.5)
ECOG		
0	78 (97.5)	72 (97.3)
1	1 (1.2)	0 (0.0)
2	1 (1.2)	2 (2.7)
CEA (ng/ml)		
Median [IQR]	2.56 [1.25‐5.70]	3.95 [1.24‐9.49]
CA 19–9 (U/ml)		
Median [IQR]	153.21 [17.35‐5318.18]	397.34 [38.97‐7105.51]
AJCC stage		
II	1 (1.2)	5 (6.8)
III	33 (41.2)	36 (48.6)
IV	46 (57.5)	33 (44.6)
Metastasis		
None	33 (38.8)	31 (41.9)
Liver	39 (45.9)	29 (39.2)
Other organs, except liver	13 (15.3)	14 (18.9)
Treatment		
Operation after CTx		
NO	67 (83.8)	68 (91.9)
YES	13 (16.2)	6 (8.1)
Chemotherapy only		
NO	1 (1.2)	1 (1.4)
YES	79 (98.8)	73 (98.6)

Abbreviations: AJCC, American Joint Committee on Cancer; BMI, body mass index; CA 19–9, carbohydrate antigen 19–9; CEA, Carcinoembryonic antigen; CTx, Chemotherapy. ECOG, Eastern Cooperative oncology Group; SD, standard deviation.

### 3.2 Evaluation of immunoprofile markers in pancreaticobiliary tract cancer tissues

**FIGURE 1:**
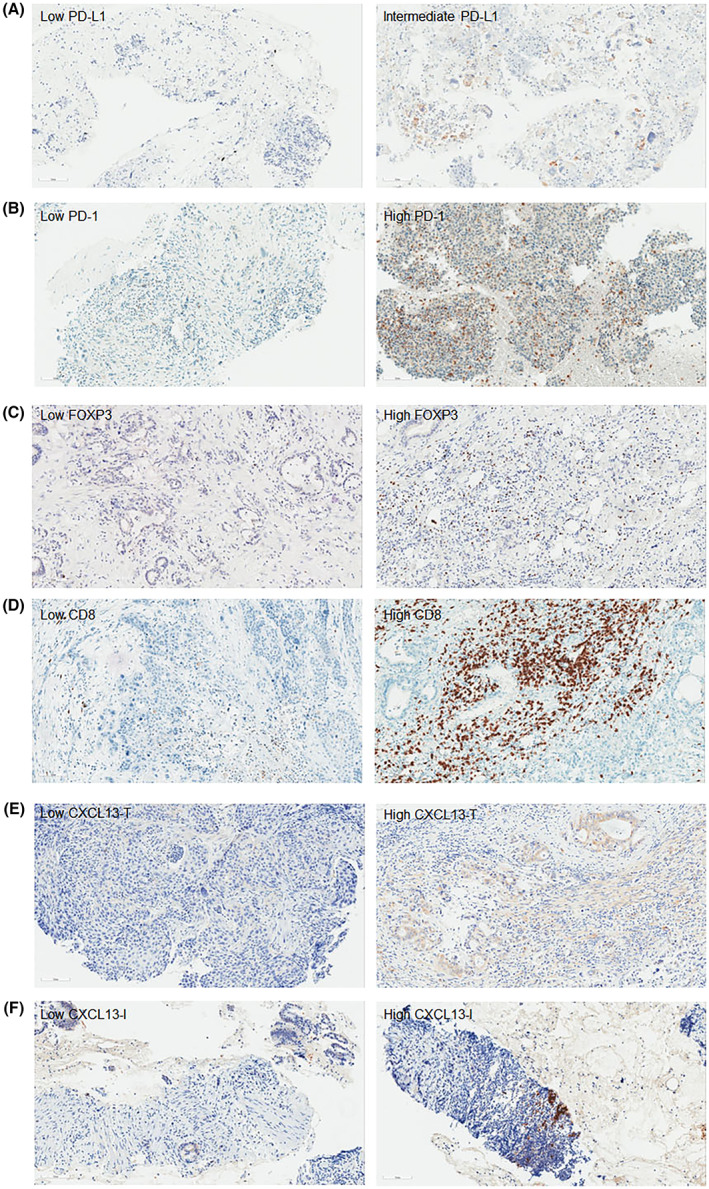
Immunohistochemical staining of immunoprofiling markers in pancreatic ductal adenocarcinoma (PDAC). The low and high expression of (A) PD‐L1, (B) PD‐1, (C) FOXP3, (D) CD8, (E) CXCL13 in tumor cells (CXCL13‐T) and (F) CXCL13 in immune cells (CXCL13‐I) in PDAC tissues were examined by immunohistochemistry. Scale bar, 100 μm

**FIGURE 2:**
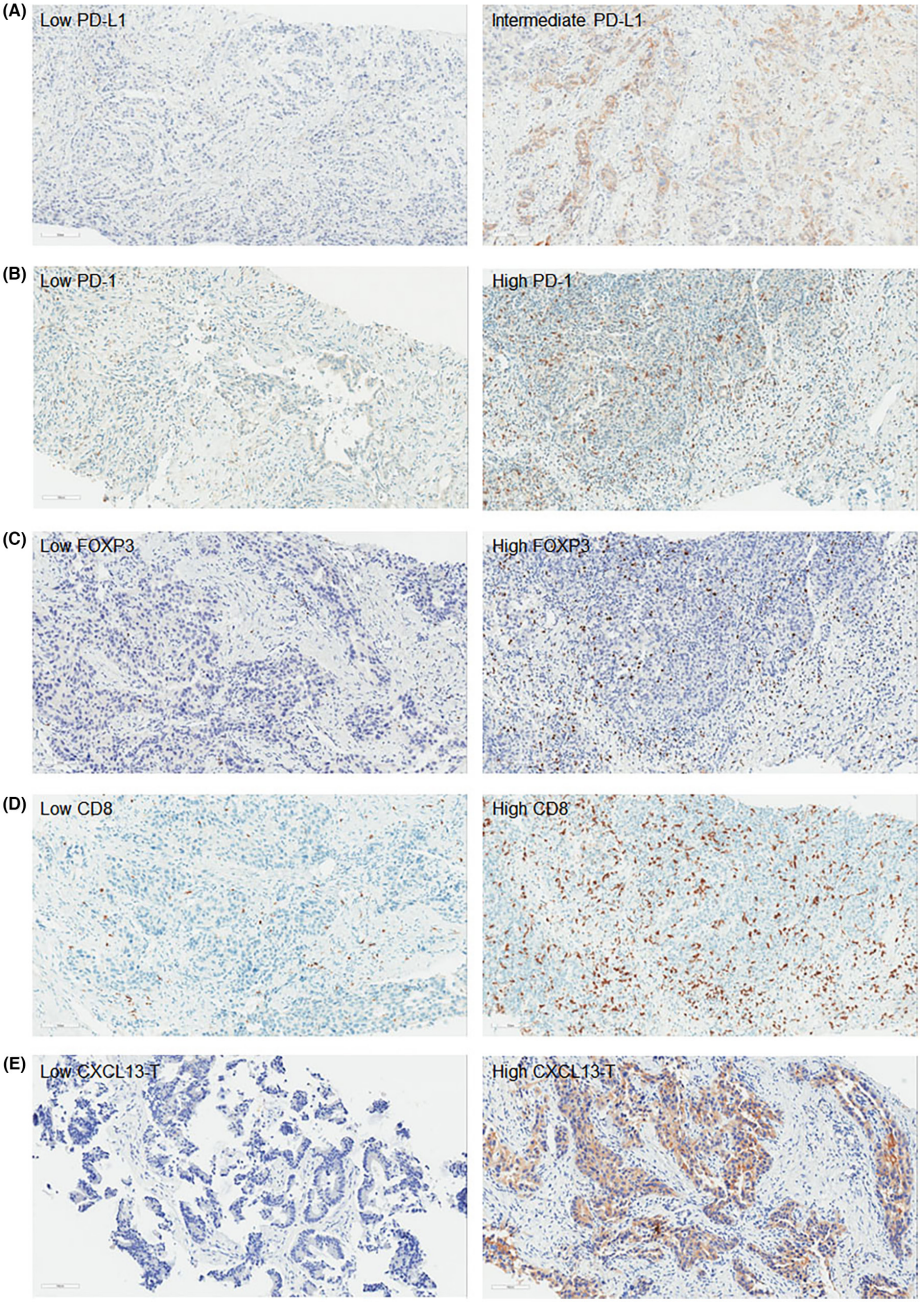
Immunohistochemical staining of immunoprofiling markers in biliary tract cancer (BTC). The low and high expression of (A) PD‐L1, (B) PD‐1, (C) FOXP3, (D) CD8, and (E) CXCL13 in BTC tissues were examined by immunohistochemistry. Scale bar, 100 μm.

We examined the expression of PD‐L1, PD‐1, FOXP3, CD8, and CXCL13 in PDAC (Figure 1) and BTC tissues (Figure 2). PD‐L1 was expressed on the membrane of tumor cells (Figure S1). Depending on the expression of PD‐L1, it was divided into three groups: low PD‐L1, less than 1%; intermediate, more than 1% but less than 50%; and high, more than 50% (Figures 1A and 2A). High PD‐L1 expression was found in only one patient with PDAC (data not shown). We divided the expression of PD‐1 into positive and negative groups depending on its expression intensity and positivity (Figures 1B and 2B). Tregs were marked with FOXP3, and the high FOXP3^+^ group was separated from the low FOXP3^+^ group based on FOXP3 counts of 20 (Figures 1C and 2C). Also, patients with CD8^+^ cells under 100 were involved in low CD8^+^ group, and patients with CD8^+^ cells over 100 belonged to high CD8^+^ group (Figures 1D and 2D). CXCL13 was expressed in both tumor cells (CXCL13‐T, Figures 1E and 2E) and immune cells in TME (CXCL13‐I, Figure 1F). The staining intensity and positive cells varied depending on specimens, which divided patients into the low CXCL13 group and high CXCL13 group (Figures 1E,F and 2E).

### 3.3 Assessment of immunoprofile markers with survival of PDAC patients

**FIGURE 3:**
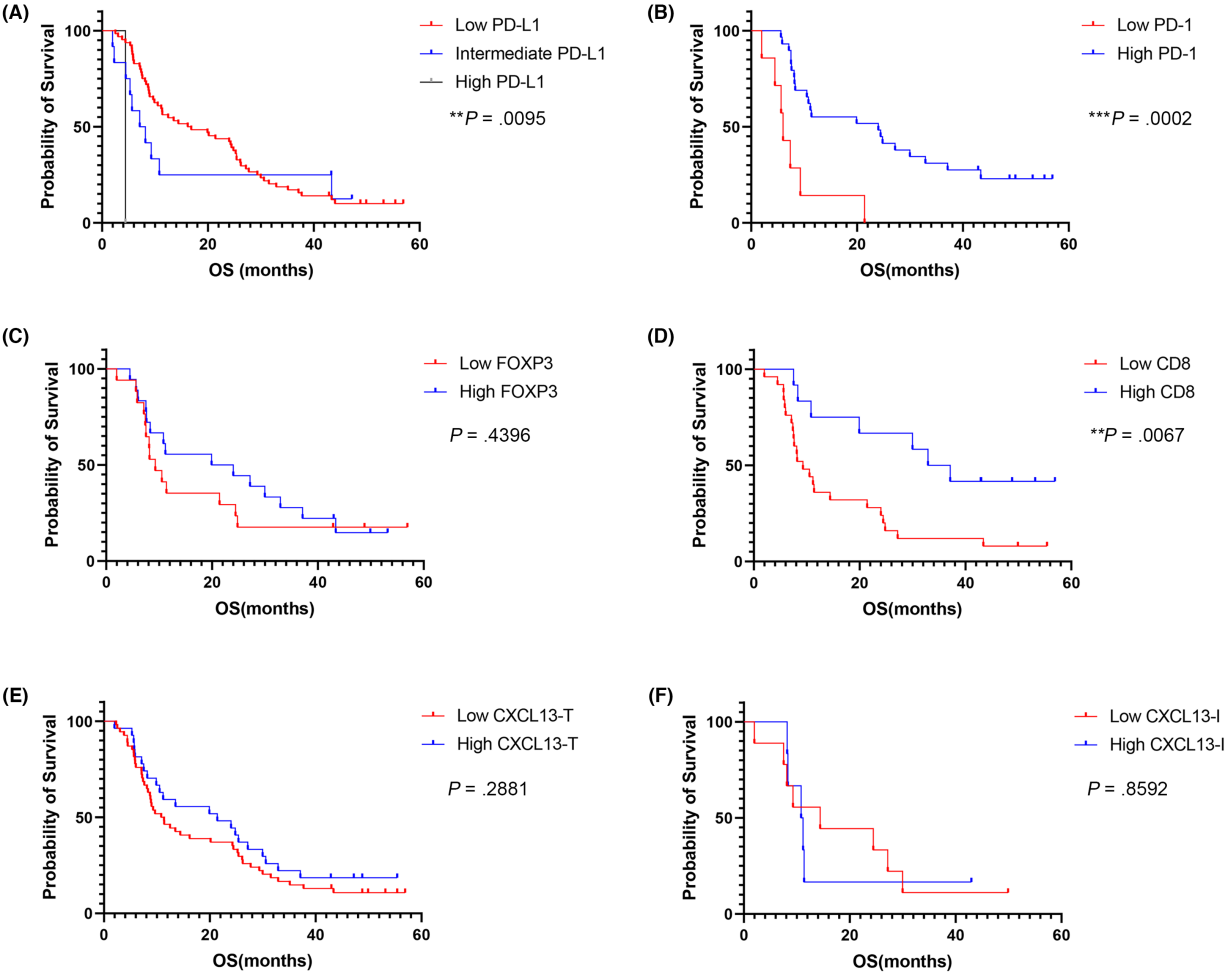
Kaplan–Meier analysis of immunoprofiling markers in pancreatic ductal adenocarcinoma (PDAC). The expression of (A) PD‐L1, (B) PD‐1, (C) FOXP3, (D) CD8, (E) CXCL13 in tumor cells (CXCL13‐T) and (F) CXCL13 in immune cells (CXCL13‐I) were analyzed with overall survival (OS) of patients with PDAC by Kaplan–Meier analysis.

Immunosuppressive TME is critically involved in prognosis and treatment response in various cancers.^17^ The median survival of the low, intermediate, and high PD‐L1 group was 16.51, 7.65, and 4.4 months, respectively. Low expression of PD‐L1 was associated with longer survival in PDAC (*p* = 0.0095, Figure 3A). Median OS (mOS) was higher in the high PD‐1 group (24.0 months) compared to the low PD‐1 group (6.01 months). High infiltration of PD‐1^+^ cells in PDAC tissues was significantly related to good prognosis (*p* = 0.0002, Figure 3B). Regarding to Treg, mOS was 9.27 months for the low FOXP3^+^ cell infiltration group and 21.9 months for the high FOXP3^+^ cell infiltration group. The infiltration of FOXP3^+^ cells could not predict the survival of PDAC patients (*p* = 0.44, Figure 3C). For CD8, the low CD8 group showed significantly shorter survival than the high CD8 group (mOS 9.27 vs. 35.0 months, *p* = 0.0067, Figure 3D). Thus, high infiltration of CD8^+^ cells was associated with a good prognosis. The expression of CXCL13 in tumor cells (CXCL13‐T, *p* = 0.288) as well as the infiltration of CXCL13^+^ cells (CXCL13‐I, *p* = 0.859) showed no significant association with the prognosis in PDAC (Figure 3E,F). Although the survival of the high CXCL13 group (mOS, 21.4 months) showed a tendency to be better than that in the low CXCL13 group (mOS, 11.9 months), the difference between the two groups was not statistically significant (Figure 3E).

### 3.4 Assessment of immunoprofile markers with survival of BTC

**FIGURE 4:**
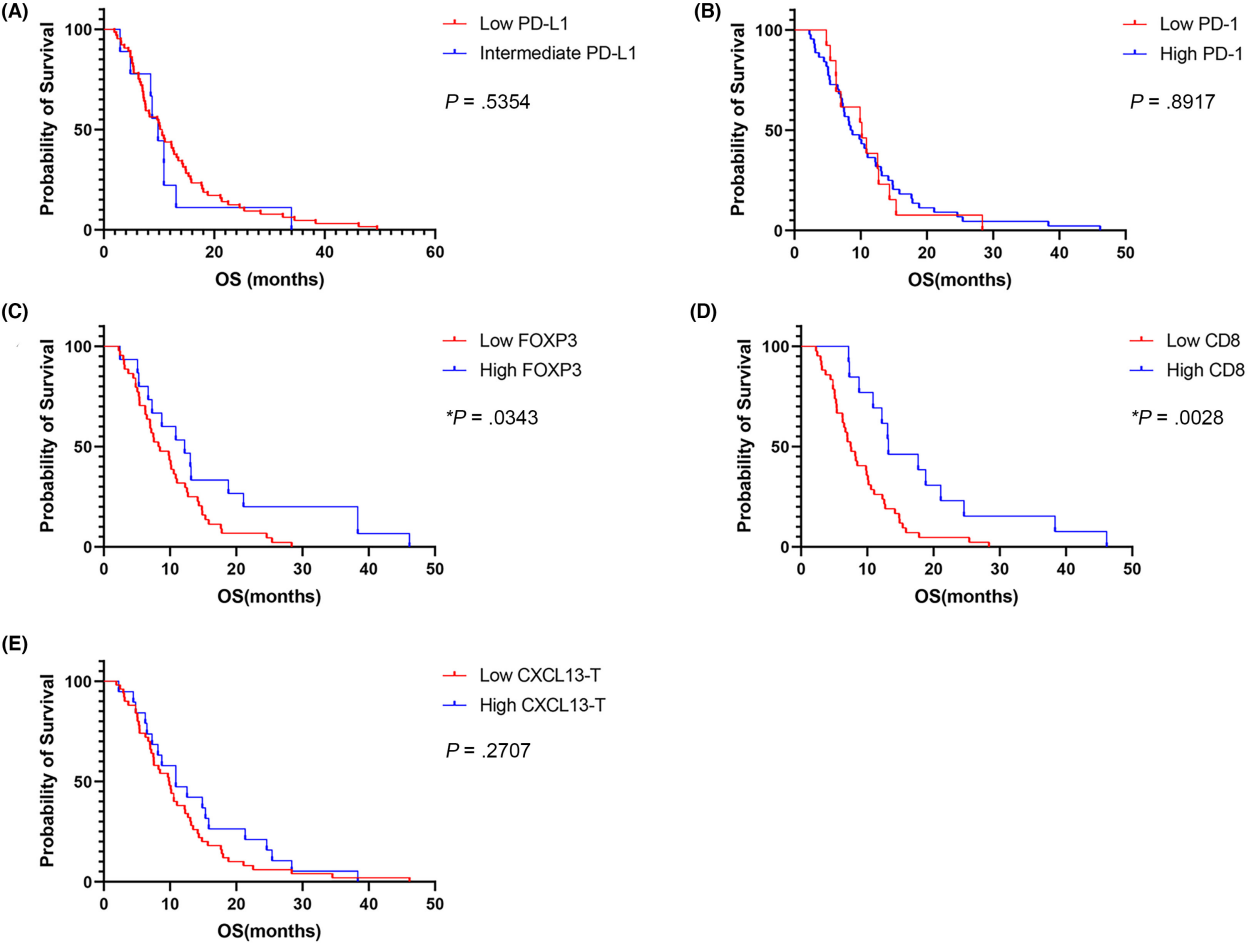
Kaplan–Meier analysis of immunoprofiling markers in biliary tract cancer (BTC). The expression of (A) PD‐L1, (B) PD‐1, (C) FOXP3, (D) CD8, and (E) CXCL13 were analyzed with overall survival (OS) of patients with BTC by Kaplan–Meier analysis.

In BTC, the median survival of low PD‐L1 group was 10.38 months, and that of high PD‐L1 group was 9.82 months. No significance was found between two groups for OS (*p* = 0.535, Figure 4A). Also, the survival of low and high PD‐1 groups was not significantly different in BTC (mOS, 8.75 vs. 10.18 months, *p* = 0.891, Figure 4B). However, patients with an increased infiltration of FOXP3^+^ cells showed longer OS in BTC (mOS, 12.21 vs. 8.48 months, *p* = 0.0343, Figure 4C). Moreover, less infiltrated CD8^+^ cells were significantly related to unfavorable survival (7.53 vs. 13.18 months, *p* = 0.0028, Figure 4D) like PDAC. Because all assessed BTC tissues were determined to have all the same pathological score one for CXCL13 expression (Figure S2) on immune cells, its relationship with survival was not analyzed. Regarding CXCL13 in tumor cells, there was no difference in OS between patients with low CXCL13 expression (mOS, 9.86 months) and patients with high CXCL13 expression (mOS, 10.88 months; *p* = 0.270, Figure 4E).

### 3.5 Assessment of immunoprofile markers upon metastasis

We also evaluated each marker with metastasis. In PDAC, CD8^+^, FOXP3^+^, and PD‐1^+^ cell infiltrations were significantly low in patients with metastasis (*p* = 0.0148, *p* = 0.0208, and *p* = 0.0318, respectively). (Figure S3A). In BTC, only FOXP3 showed significant association with metastasis (*p* = 0.005), but CD8, PD‐1, PD‐L1, and CXCL13 did not (Figure S3B). Moreover, we evaluated the expression of each marker in primary pancreatic tumor and metastatic liver in PDAC (Figure S3C). The infiltration of CD8^+^, FOXP3^+^, and PD‐1^+^ cells were higher in primary pancreas in compared to liver metastases (*p* = 0.0302, *p* = 0.0522 and *p* = 0.0296, respectively), and the expression of CXCL13 was significantly increased in metastatic liver (*p* = 0.0337). We could not examine metastatic sites in BTC.

### 3.6 Correlation between infiltrated immune cells based on immunoprofile markers

In TME, the relationship of infiltrated immune cells was assessed by Pearson's correlation coefficient. In PDAC (Figure 4A), there was a significant correlation of infiltrated CD8^+^ T cells and FOXP3^+^ Tregs (*r* = 0.5533, *p* = 0.0006), PD‐1^+^ cells and FOXP3^+^ Tregs (*r* = 0.4779, *p* = 0.0037), and PD‐1^+^ cells and CD8^+^ T cell (*r* = 0.7121, *p* < 0.0001). In BTC (Figure 4B), we also confirmed a positive correlation between infiltrated PD‐1^+^ cells and cytotoxic CD8^+^ T cells (*r* = 0.7541, *p* < 0.0001), between PD‐1^+^ cells and CD8^+^ T cells (*r* = 0.6706, *p* < 0.0001), or between PD‐1^+^ cells and FOXP3^+^ Tregs (*r* = 0.7963, *p* < 0.0001). It indicates a significant relationship in the infiltration of immune cells in PDAC and BTC.

### 3.7 Analysis of clinical factors and immunoprofiles with OS

We evaluated clinical factors and immunoprofiling markers for survival in PDAC (Table 2). In univariate analysis, significant clinical value was operation after chemotherapy (*p* < 0.001), mass size (*p* = 0.0004), and metastasis (*p* = 0.006). Among immunoprofiling markers, the significant factors were PD‐L1 (*p* < 0.0001), PD‐1 (*p* = 0.003), and CD8 (*p* = 0.0063). Multivariable analysis found operation after chemotherapy (*p* = 0.021) as prognostic markers in PDAC.

**TABLE 2:** Analysis of clinical factors and immunoprofiles with overall survival in pancreatic ductal adenocarcinoma

Clinical Factor	Univariate analysis (n = 100, event = 87)		Multivariate analysis (n = 26, event = 20)	
	HR (95% CI)	*p*‐value	HR (95% CI)	*p*‐value
Age (years)	0.99 (0.97–1.02)	0.76		
Sex				
Female				
Male	1.24 (0.75–2.05)	0.40		
BMI	1.01 (0.95–1.08)	0.75		
ECOG		0.56		
0	Ref			
1	1.08 (0.78–1.48)	0.64		
2	1.05 (0.76–1.44)	0.76		
Smoking		0.069		
Never	Ref			
Former	0.85 (0.43–1.66)	0.63		
Current	1.99 (1.05–3.78)	0.025		
CEA (*n* = 66)	1.02 (1.00–1.04)	0.027	0.98 (0.95–0.99)	0.025
CA 19–9 (*n* = 97)	1	<0.0001	1	0.026
Operation after CTx				
NO	Ref		Ref	
YES	0.18 (0.07–0.42)	<0.0001	0.21 (0.05–0.79)	0.021
Mass size	1.31 (1.13–1.53)	0.0004		
Stage		<0.0001		
II	Ref			
III	0.04 (0.02–0.09)	<0.0001	Ref	
IV	0.08 (0.04–0.18)	<0.0001	3.77 (0.62–22.72)	0.14
Metastasis		0.006		
None	ref			
Liver	1.59 (0.74–3.43)	0.23		
Other organs	1.01 (0.55–1.85)	0.96		
Both	3.15 (1.55–6.38)	0.001		

**IHC marker**	**Univariate analysis** **HR (95% CI)**	***p*‐value**	**Multivariate analysis** **HR (95% CI)**	***p*‐value**
PD‐L1 (*n* = 95)		<0.0001		
PD‐L1 < 1%	Ref		Ref	
1% ≤ PD‐L1 < 50%	1.57 (0.65–3.77)	0.31	1.95 (0.57–6.71)	0.29
PD‐L1 ≥ 50%	16.15 (7.12–36.64)	<0.0001	‐	
PD‐1 (*n* = 36)		0.003		0.096
PD‐1 < 2	Ref		Ref	
2 ≤ PD‐2 < 20	0.36 (0.15–0.86)	0.022	0.27 (0.08–0.90)	0.033
PD‐1 ≥ 20	0.17 (0.06–0.47)	0.0007	0.84 (0.08–8.74)	0.88
FOXP3 (*n* = 35)		0.76		
FOXP3 < 4	Ref			
4 ≤ FOXP3 < 30	0.67 (0.16–2.79)	0.57		
FOXP3 ≥ 30	0.59 (0.15–2.42)	0.47		
CD8 (*n* = 37)		0.0063		0.74
CD8 < 100	Ref		Ref	
100 ≤ CD8 < 200	0.44 (0.11–1.71)	0.23	1.11 (0.13–9.14)	0.92
CD8 ≥ 200	0.26 (0.11–0.61)	0.002	0.53 (0.10–2.72)	0.44
CXCL13 (*n* = 15)		0.21		
CXCL13 < 100	Ref			
100 ≤ CXCL13 < 200	2.20 (0.73–6.65)	0.16		
CXCL13 ≥ 200	0.66 (0.14–3.27)	0.61		
CXCL13T (*n* = 92)		0.29		
CXCL13 < 1	Ref			
1 ≤ CXCL13 < 50	0.78 (0.49–1.23)	0.28		
CXCL13 ≥ 50	0.69 (0.39–1.20)	0.19		

*Note*: Patients were grouped divided by mean values for each immune marker.

Abbreviations: PD‐1, Programmed cell death protein‐1; PD‐L1, positive programmed death‐ligand 1.

For BTC (Table 3), ECOG (*p* = 0.0002) showed significant value among clinical factors by univariate analysis. Among immunoprofiling markers, CD8 was found to show significant value in both univariate (*p* = 0.007) and multivariate (*p* = 0.037) analysis.

**TABLE 3:** Analysis of clinical factors and immunoprofiles with overall survival in biliary tract cancer

Clinical factor	Univariate analysis		Multivariate analysis (*n* = 45, event = 45)	
	HR (95% CI)	*p*‐value	HR (95% CI)	*p*‐value
Age (years)	1.01 (0.98–1.03)	0.58		
Sex				
Female	Ref			
Male	0.75 (0.45–1.26)	0.28		
BMI	1.09 (0.99–1.19)	0.052		
ECOG				
0	Ref			
1	‐			
2	6.84 (2.48–18.92)	0.0002		
Smoking		0.067		
Never	Ref			
Former	0.69 (0.39–1.21)	0.19		
Current	0.43 (0.20–0.92)	0.030		
CEA (*n* = 60)	1.00 (1–1.01)	0.029	1.00 (0.99–1.01)	0.37
CA 19–9 (*n* = 81)	1	0.11		
Operation after CTx				
NO	Ref			
YES	0.39 (0.15–1.09)	0.074		
Mass size	0.02 (0.95–1.08)	0.52		
Stage		0.056		
II	Ref			
III	1.76 (0.54–5.78)	0.35		
IV	2.87 (0.89–9.23)	0.077		
Metastasis		0.70		
None	Ref			
Liver	1.19 (0.59–2.35)	0.62		
Other organs	1.01 (0.42–2.37)	0.99		
Both	1.47 (0.75–2.87)	0.25		

**IHC marker**	**Univariate analysis** **HR (95% CI)**	***p*‐value**	**Multivariate analysis** **HR (95% CI)**	***p*‐value**
PD‐L1 (*n* = 79)				
PD‐L1 < 1%	Ref			
1% ≤ PD‐L1 < 50%	1.31 (0.68–2.52)	0.41		
	‐			
PD‐1 (*n* = 55)		0.41		
PD‐1 < 2	Ref			
2 ≤ PD‐2 < 20	0.97 (0.56–1.71)	0.94		
PD‐1 ≥ 20	0.63 (0.29–1.39)	0.26		
FOXP3 (*n* = 58)		0.72		
FOXP3 < 4	Ref			
4 ≤ FOXP3 < 30	1.25 (0.64–2.46)	0.51		
FOXP3 ≥ 30	0.92 (0.42–2.04)	0.83		
CD8 (*n* = 58)		0.007		0.037
CD8 < 100	Ref		Ref	
100 ≤ CD8 < 200	0.40 (0.23–0.71)	0.0016	0.39 (0.18–0.82)	0.013
CD8 ≥ 200	0.85 (0.40–1.84)	0.69	1.04 (0.38–2.78)	0.94
CXCL13				
CXCL13 < 100		NA		
100 ≤ CXCL13 < 200		NA		
CXCL13 ≥ 200		NA		
CXCL13T (*n* = 75)		0.50		
CXCL13 < 1	Ref			
1 ≤ CXCL13 < 50	0.71 (0.39–1.30)	0.26		
CXCL13 ≥ 50	0.71 (0.35–1.43)	0.33		

Abbreviations: AJCC, American Joint Committee on Cancer; BMI, body mass index; CA 19–9, carbohydrate antigen 19–9; CEA, Carcinoembryonic antigen; CTx, Chemotherapy; ECOG, Eastern Cooperative Oncology Group; PD‐1, Programmed cell death protein‐1; PD‐L1, positive programmed death‐ligand 1; SD, standard deviation.

### 3.8 Analysis of the association of clinical factors and immunorprofiles with treatment response

Prognostic factors for response to cancer treatment were determined. It was assessed clinical factors and immunoprofiling factors depending on the progress after the first chemotherapy. In univariable analysis, ECOG (*p* < 0.0001), CD8 (*p* = 0.036), and CXCL13 in tumor cells (*p* < 0.0001) were critical prognostic markers for disease progression after treatment in PDAC (Table S2). By multivariable analysis, ECOG status (*p* = 0.039), and CD8 (*p* = 0.038) were confirmed as prognostic markers for response to chemotherapy. The expression of CXCL13 in tumor cells was also significantly associated with treatment response (*p* = 0.043; Table S2).

In BTC, prognostic factors affecting response to the first chemotherapy were operation after chemotherapy and tumoral CXCL13 expression in both univariate analysis (*p* = 0.023 and *p* < 0.0001, respectively) and multivariate analysis (*p* < 0.0001 and *p* = 0.003, respectively; Table S3).

## 4 DISCUSSION

In this study, we aimed to predict the survival and find prognostic factors at the time of diagnosis of pancreaticobiliary tract cancer. It was found that the expression of PD‐L1, PD‐1, and CD8 is related to survival and the expression of CD8, FOXP3, and PD‐1 is associated with metastasis in PDAC patients. Moreover, CD8 and FOXP3 were prognostic markers for survival, and FOXP3 expression was a marker connected to metastasis in BTC.

The prognosis of pancreatic cancer and biliary tract cancer is very poor.^18^, ^19^ Thus, it is important to determine prognostic factors in advance at the time of diagnosis and predict them to determine the treatment policy. A previous study using whole‐exome sequencing and in silico neoantigen prediction has found that tumors with both the highest neoantigen number and the most abundant CD8^+^ T‐cell infiltrates, but not with one of them alone, could stratify patients with the longest survival.^1^ A meta‐analysis identified prognostic factors for tumor infiltrating lymphocytes in pancreatic cancer, and CD8 was found to be associated with good OS while FOXP3 was associated with poor prognosis.^20^ In our study, high level of CD8^+^ cells were related to longer OS in both PDAC and BTC. FOXP3‐expressing Treg cells, which suppress aberrant immune response against self‐antigens, also can suppress anti‐tumor immune response.^9^ Infiltration of a large number of Treg cells into tumor tissues is often associated with poor prognosis,^21^, ^22^ which was concordant with our result that infiltrations of FOXP3^+^ cells were associated with good prognosis in BTC. However, we found a positive correlation between CD8^+^ cells and FOXP3^+^ cells, indicating the importance of infiltrated T cells for better survival.

CXCL13 has been reported as a prognostic factor in gastric cancer.^23^ Although it was not significantly associated with OS in both PDAC and BTC, we confirmed to be a significant prognostic factor in disease progression after chemotherapy. However, we investigated responses only to the first regimen of chemotherapy and further studies fully considering all chemotherapy regimens and their responses are needed. Many clinical trials targeting immune checkpoint regulators, PD‐1 and PD‐L1, showed promising results in various hematologic and solid cancers.^24^ PD‐1 is expressed on the surface of macrophages or lymphocytes and rarely expressed in non‐neoplastic epithelial tissues. Cancer cells can express PD‐L1 to suppress the host's anti‐tumor immune response and escape it.^25^ In a previous study, higher PD‐L1 expression was significantly correlated with better overall survival and disease‐free survival in periampullary/pancreatic cancer patients.^26^ In another study, PD‐L1 positivity had a poor prognosis.^12^ PD‐L1/PD‐1 pathway may be a critical regulator in human pancreatic cancer. Because PD‐L1 expression was inversely correlated with tumor‐infiltrating T lymphocytes, particularly CD8^+^ T cells. Blocking PD‐L1 promotes infiltrative CD8 penetration, and there is also a study showing a synergistic effect when treated with a blocking agent and Gemcitabine. As proven, a combination of anti–PD‐L1 monoclonal antibody and gemcitabine exhibited a significant synergistic effect on murine pancreatic cancer.^12^ Therefore, if the prognosis is confirmed and leads to the development of a treatment, it could be a breakthrough discovery. In another study, high expression of PD‐L1 on cancer cell membranes correlated with lymph node metastasis and strongly correlated with poor differentiation. Like most previous studies, the expression PD‐L1 was related to the poorer survival in PDAC. However, PD‐L1 expression of BTC tissues was not associated with prognosis in our results. There are not many studies on PD‐1^+^ cell and prognosis in pancreaticobiliary cancer. A report showed that high proportion of tumor‐infiltrating CD8^+^ PD‐1^High^ T cells significantly correlated with advanced tumor‐node‐metastasis (TNM) stage and worse postoperative survival in intrahepatic cholangiocarcinoma.^27^ Besides, the expression of PD‐L1 was correlated with the expression of PD‐1, and the expression of PD‐L1 and PD‐1 was significantly correlated with TMN stage, lymphatic metastasis, and the survival time of patients in extrahepatic cholangiocarcinoma.^28^ Our study is a very new attempt because it not only confirmed the prognosis value of various profiles but also analyzed both pancreatic and biliary tract cancers.

This study has some limitations. As a retrospective study, limited clinical factors were collected by reviewing electronic medical records. Because not all specimens were stored in the pathology department, each IHC marker could not be tested in all patients. Also, the number of PDAC and BTC tissue samples that we can collect and the number of examined factors that we can analyze were not enough to have stronger statistical power. Another limitation is that patients have the right to get treatment and most of them undergo chemotherapy, so a perfect hypothesis for a naive prognostic marker cannot be established. Moreover, this was a single center study. We need another validation set to prove each IHC marker in PDAC and BTC.

In conclusion, IHC markers that can influence the survival rate in pancreaticobiliary tract cancer are FOXP3 or CD8 in BTC, and PD‐L1, PD‐1, or CD8 in PDAC. A high expression of CXCL13 was related to good response to chemotherapy in BTC. Further studies are needed to develop and introduce therapeutic agents by identifying novel markers and enhancing their immunity.

## AUTHOR CONTRIBUTIONS

**Ji Eun Kim:** Data curation (equal); formal analysis (equal); writing – original draft (equal). **Hyemin Kim:** Data curation (supporting); formal analysis (supporting); software (supporting). **Binnari Kim:** Resources (equal). **Hye Gyo Chung:** Data curation (supporting). **Hwe Hoon Chung:** Resources (supporting). **Kyoung Mee Kim:** Resources (supporting). **Seong Hyun Kim:** Resources (supporting). **Woo Kyoung Jeong:** Resources (supporting). **Young Kon Kim:** Resources (supporting). **Ji Hye Min:** Resources (supporting). **Jin Seok Heo:** Resources (supporting). **In Woong Han:** Resources (supporting). **Sang Hyun Shin:** Resources (supporting). **Hee Chul Park:** Resources (supporting). **Jeong Il Yu:** Resources (supporting). **Joon Oh Park:** Resources (supporting). **Seung Tae Kim:** Resources (supporting). **Jung Yong Hong:** Resources (supporting). **Se‐Hoon Lee:** Resources (supporting). **Kwang Hyuk Lee:** Resources (supporting). **Jong Kyun Lee:** Resources (supporting). **Kyu Taek Lee:** Writing – review and editing (lead). **Kee‐Taek Jang:** Resources (lead). **Joo Kyung Park:** Writing – review and editing (lead).

## FUNDING INFORMATION

This work has supported by grants of the National Research Foundation (NRF) funded by the Ministry of Science and ICT (MSIT), Republic of Korea (Gant No. NRF‐2019R1C1C1008646, NRF‐2020R1A2C2102023). It was also supported by the Korean Gastroenterology Fund for Future Development. This study was supported by Future Medicine 20*30 project of the Samsung Medical Center (SMC) (Gant No. SMX 1220091, SMX1220111) and SMC Research and Development Grant (Gant No. SMO1220261).

## CONFLICT OF INTEREST

The authors declare that the research was conducted in the absence of any commercial or financial relationships that could be construed as a potential conflict of interest.

## PATIENT CONSENT FOR PUBLICATION

Not required.

## ETHICS APPROVAL

Ethical approval was obtained from the Institutional Review Board (IRB) of Samsung Medical Center (IRB No.2020–07‐012).

## PROVENANCE AND PEER REVIEW

Not commissioned; externally peer reviewed.

## NOVELTY AND IMPACT

IHC markers that can influence the survival rate in pancreaticobiliary tract cancer are FOXP3 or CD8 in BTC, and PD‐L1, PD‐1, or CD8 in PDAC. A high expression of CXCL13 was related to good response to chemotherapy in BTC. Further studies are needed to develop and introduce therapeutic agents by identifying novel markers and enhancing their immunity.

## Supporting information


Data S1.

## Data Availability

The complete dataset is available from the corresponding author upon reasonable request.
