# Supplementary material for: Comprehensive immunoprofile analysis of prognostic markers in pancreaticobiliary tract cancers
Source: Cancer Med. 2023 Jan 17;12(7):7748–61. doi: 10.1002/cam4.5530 (PMC10134292; doi:10.1002/cam4.5530)
Supplement: Supplementary file 1 — Data S1. [file CAM4-12-7748-s001.docx]

Supplementary Material

Comprehensive immunoprofile analysis of prognostic markers in pancreaticobiliary tract cancers

Ji Eun Kim^1,†^ | Hyemin Kim^1,2,†^ | Binnari Kim^5,†^ | Hye Gyo Chung^1^ | Hwe Hoon Chung^1^ | Kyoung Mee Kim^4^, Seong Hyun Kim^6^ | Woo Kyoung Jeong^6^ | Young Kon Kim^6^ | Ji Hye Min^6^ | Jin Seok Heo^7^ | In Woong Han^7^ | Sang Hyun Shin^7^ | Hee Chul Park^8^ | Jeong Il Yu^8^ | Joon Oh Park^9^ | Seung Tae Kim^9^ | Jung Yong Hong^9^ | Se-Hoon Lee^9^ | Kwang Hyuck Lee^1^ | Jong Kyun Lee^1^ | Kyu Taek Lee^1,^* | Kee-Taek Jang^4,^* | Joo Kyung Park^1,3,^*

^1^Department of Medicine, Samsung Medical Center, Sungkyunkwan University School of Medicine, Seoul, Korea.

^2^Medical Research Institute, Sungkyunkwan University School of Medicine, Seoul, Korea.

^3^Department of Health Sciences and Technology, SAIHST, Sungkyunkwan University, Seoul, Korea

^4^Department of Pathology, Samsung Medical Center, Sungkyunkwan University School of Medicine, Seoul, Korea.

^5^Department of Pathology, Ulsan University Hospital, University of Ulsan College of Medicine, Ulsan, Korea.

^6^Department of Radiology, Samsung Medical Center, Sungkyunkwan University School of Medicine, Seoul, Korea.

^7^Department of Hepato Biliary Pancreatic Surgery, Samsung Medical Center, Sungkyunkwan University School of Medicine, Seoul, Korea.

^8^Department of Radiation Oncology, Samsung Medical Center, Sungkyunkwan University School of Medicine, Seoul, Korea.

^9^Department of Hematology/Oncology

## Supplementary Figures


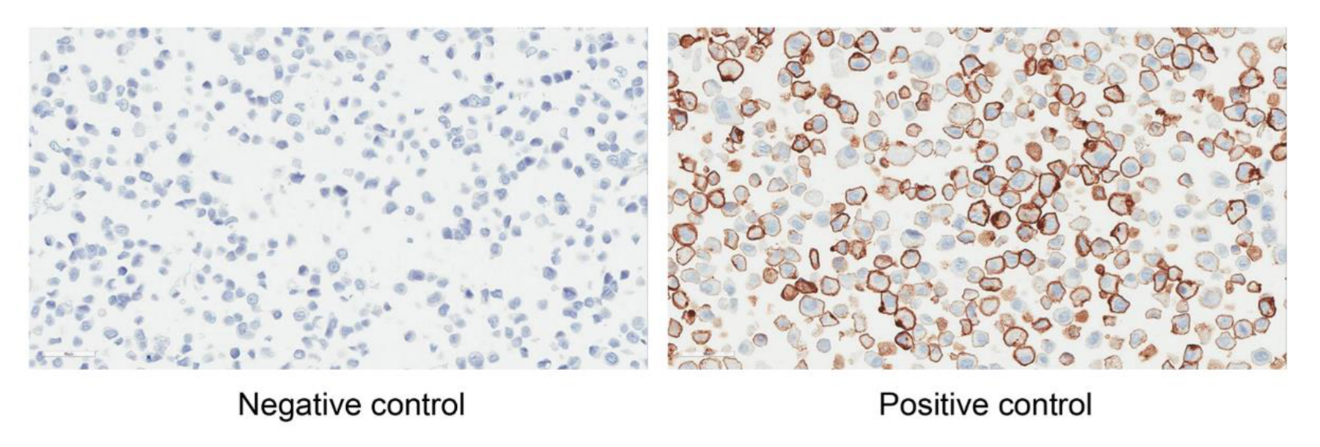


**Supplementary Figure 1**  Positive and negative controls for PD-L1 expression.

We tested anti-PD-L1 (22C3) antibody for immunohistochemistry by using tonsil tissues. Scale bar, 50 μm.

**
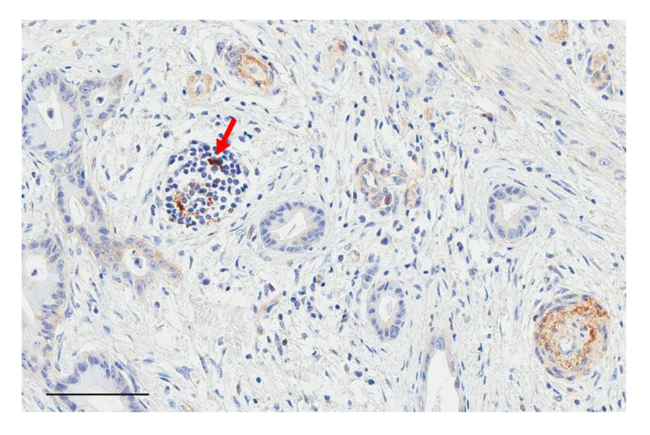
**

**Supplementary Figure 2** CXCL13 expression in BTC.

The expression of CXCL13 was examined with immunohistochemistry and the expression level was scored in BCT tissues. Scale bar, 100 μm.


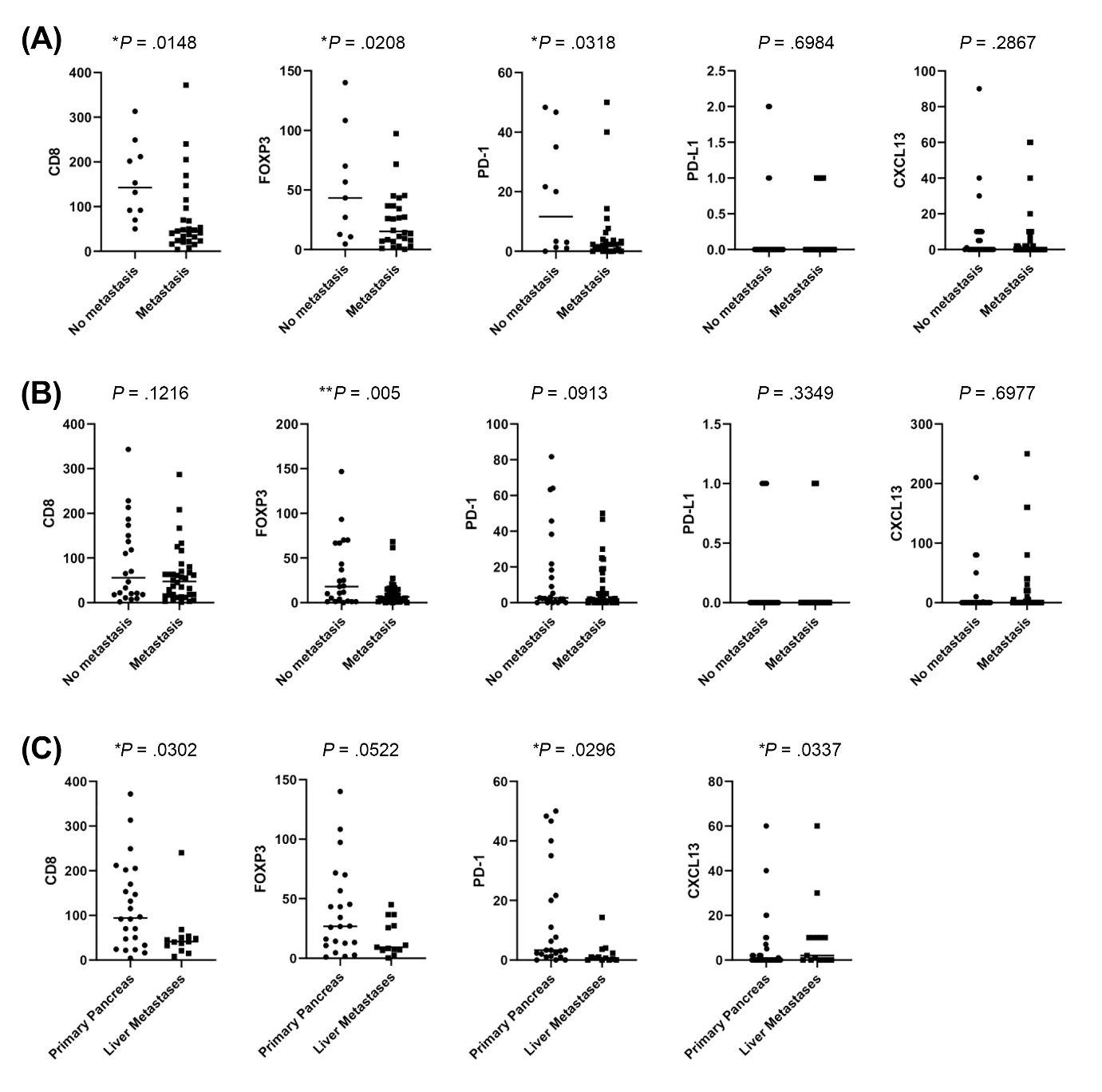


**Supplementary Figure 3** Evaluation of immunoprofiling markers with metastasis in pancreatic ductal adenocarcinoma (PDAC) and biliary tract cancer (BTC).

The expression score of CD8, FOXP3, PD-L1, PD-1, and CXCL13 was evaluated with the existence of metastasis in (**A**) PDAC and (**B**) BTC. (**C**) In PDAC, the expression score of CD8, FOXP3, PD-1, and CXCL13 was assessed in primary pancreatic tumor site and metastatic liver site.


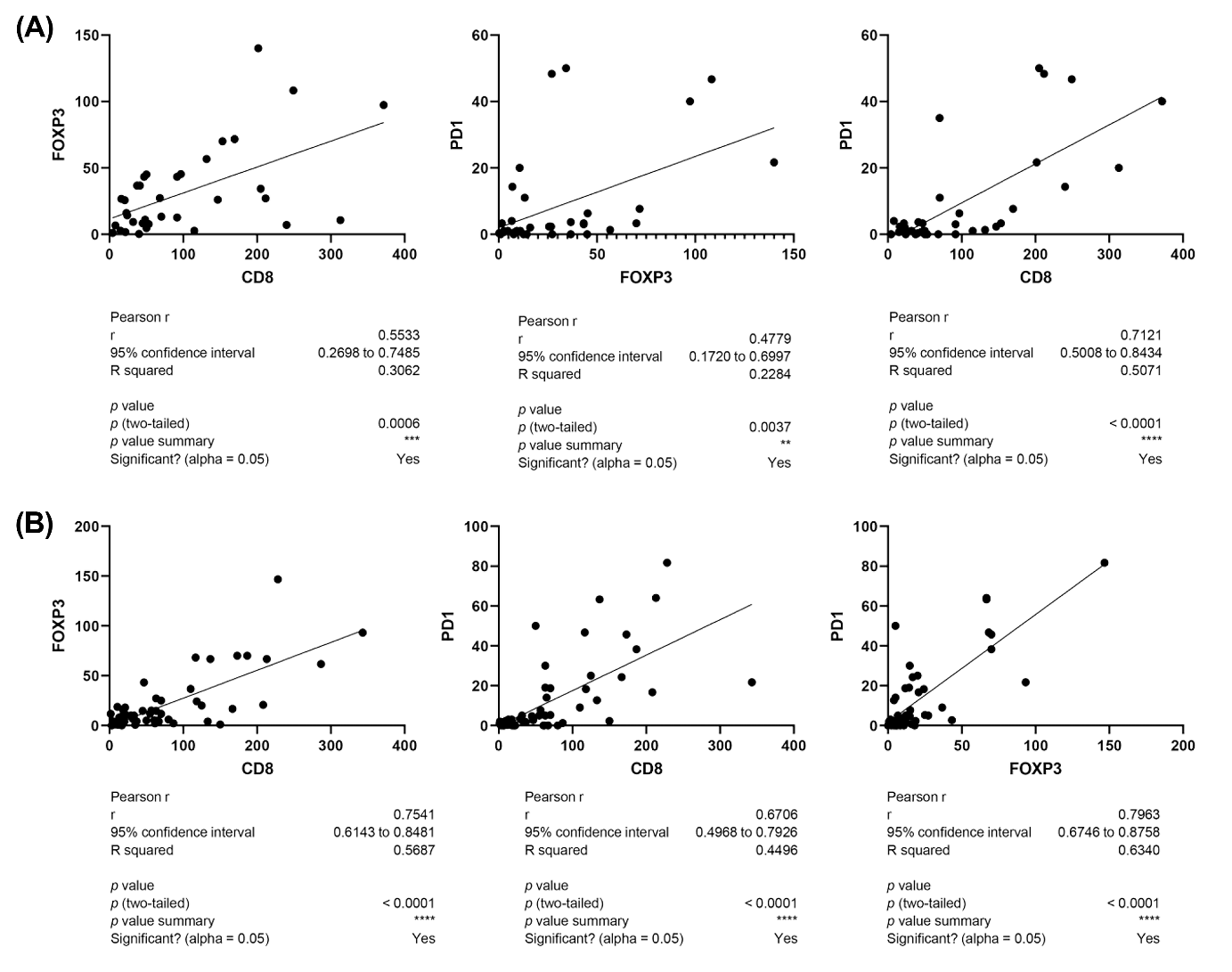


**Supplementary Figure 4** Correlation analysis of immunoprofiling markers in pancreatic ductal adenocarcinoma (PDAC) and biliary tract cancer (BTC).

Correlation of infiltrated Cytotoxic CD8^+^ T cells and FOXP3^+^ regulatory T cells (Tregs), PD-1^+^ cells and FOXP3^+^ Tregs, or PD-1^+^ cells and FOXP3^+^ Tregs in (**A**) PDAC and (**B**) BTC was analyzed by Pearson's correlation coefficient.

**Supplementary Tables**

**Supplementary Table 1** Antibody information for immunohistochemistry

| **Antibody** | **Company** | **Catalog #** | **Dilution** |
| --- | --- | --- | --- |
| PD-L1 IHC 22C3 PharmDx | DAKO Agilent | SK006 | RTU |
| PD-1 (NAT105) Mouse Monoclonal Antibody | Ventana Medical Systems, Roche | 760-4895 | RTU |
| CD8 (SP57) Rabbit Monoclonal Primary Antibody | Ventana Medical Systems, Roche | 790-4460 | RTU |
| Recombinant Anti-FOXP3 (236A/E7) antibody | Abcam | ab20034 | 1:200 |
| Human CXCL13/BLC/BCA-1 Antibody | R&D Systems | AF801 | 1:1000 |
| BOND™ Polymer Refine Detection Kit | Leica Biosystem | DS9800 | RTU |
| OptiView DAB IHC detection kit | Ventana Medical Systems, Roche | 760-700 | RTU |
| Ultraview universal DAB detection kit | Ventana Medical Systems, Roche | 760-500 | RTU |

*RTU: Ready-to-Use

**Supplementary Table 2** Analysis of clinical factors and immunoprofiles with disease progression in pancreatic ductal adenocarcinoma

| **Clinical Factor** | | **Univariate analysis**  **(n = 73, event = 16)** | | | | **Multivariate analysis 3**  **(n = 29, event = 5)** | | | |
| --- | --- | --- | --- | --- | --- | --- | --- | --- | --- |
|  | **HR (95% CI)** | | | | ***P*-value** | **HR (95% CI)** | | | ***P*-value** |
| **Age (years)** | 1.01 (0.99-1.02) | | | | .14 |  |  |  |  |
| **Sex** |  | |  |  |  |  |  |  |  |
| Female | ref | | | |  |  |  |  |  |
| Male | 0.92 (0.77-1.11) | | | | .39 |  |  |  |  |
| **BMI** | 0.99 (0.97-1.02) | | | | .84 |  |  |  |  |
| **ECOG** |  | |  |  |  |  |  |  |  |
| 0 | ref | | | |  | ref | | |  |
| 1 | - | | | |  | - |  |  |  |
| 2 | 0.80 (0.73-0.87) | | | | <.0001 | 0.74 (0.55-0.98) | | | .039 |
| **Smoking** |  | |  |  | .89 |  |  |  |  |
| Never | ref | | | |  |  |  |  |  |
| Former | 1.034 (0.84-1.27) | | | | .71 |  |  |  |  |
| Current | 1.05 (0.81-1.36) | | | | .71 |  |  |  |  |
| **CEA (n = 48)** | 0.99 (0.98-1.00) | | | | .28 |  |  |  |  |
| **CA 19-9 (n = 70)** | 1 | | | | .072 |  |  |  |  |
| **Operation after CTx** |  | |  |  |  |  |  |  |  |
| NO | ref | | | |  |  |  |  |  |
| YES | 0.88 (0.73-1.07) | | | | .21 |  |  |  |  |
| **Mass size** | 1.06 (0.99-1.12) | | | | .066 |  |  |  |  |
| **Stage** |  | |  |  |  |  |  |  |  |
| II | - | | | |  |  |  |  |  |
| III | ref | | | |  |  |  |  |  |
| IV | 0.93 (0.78-1.12) | | | | .45 |  |  |  |  |
| **Metastasis** |  | |  |  | .45 |  |  |  |  |
| None | ref | | | |  |  |  |  |  |
| Liver | 0.95 (0.76-1.19) | | | | .66 |  |  |  |  |
| Other organs | 0.85 (0.68-1.07) | | | | .17 |  |  |  |  |
| Both | 1.04 (0.80-1.34) | | | | .79 |  |  |  |  |
| **IHC marker** | **Univariate analysis**  **HR (95% CI)** | | |  | ***P*-value** | **Multivariate analysis**  **HR (95% CI)** | |  | ***P*-value** |
| **PD-L1 (n = 70)** |  | |  |  |  |  |  |  |  |
| PD-L1<1% | ref | | | |  |  |  |  |  |
| 1%≤PD-L1<50% | 1.22 (0.91-1.64) | | | | .17 |  |  |  |  |
| PD-L1≥50% | - | |  |  |  |  |  |  |  |
| **PD-1 (n = 28)** |  | |  |  | .48 |  |  |  |  |
| PD-1<2 | ref | | | |  |  |  |  |  |
| 2≤PD-2<20 | 0.91 (0.69-1.21) | | | | .53 |  |  |  |  |
| PD-1≥20 | 1.16 (0.75-1.81) | | | | .50 |  |  |  |  |
| **FOXP3 (n = 27)** |  | |  |  | .63 |  |  |  |  |
| FOXP3<4 | ref | | | |  |  |  |  |  |
| 4≤FOXP3<30 | 0.98 (0.60-1.59) | | | | .94 |  |  |  |  |
| FOXP3≥30 | 0.86 (0.54-1.37) | | | | .52 |  |  |  |  |
| **CD8 (n = 29)** |  | |  |  | .036 |  | | | .038 |
| CD8<100 | ref | | | |  | ref | | | .069 |
| 100≤CD8<200 | 0.84 (0.71-1.01) | | | | .057 | 0.84 (0.69-1.01) | | | .069 |
| CD8≥200 | 1.18 (0.78-1.79) | | | | .43 | 1.29 (0.78-2.10) | | | .31 |
| **CXCL13 (n = 11)** |  | |  |  | .17 |  |  |  |  |
| CXCL13<100 | ref | | | |  |  |  |  |  |
| 100≤CXCL13<200 | 1.39 (0.82-2.38) | | | | .22 |  |  |  |  |
| CXCL13≥200 | 1.65 (0.82-3.30) | | | | .15 |  |  |  |  |
| **CXCL13T (n = 68)** |  | |  |  | <.0001 |  |  |  | .043 |
| CXCL13<1 | ref | | | |  | ref | | |  |
| 1≤CXCL13<50 | 1.01 (0.80-1.28) | | | | .93 | 1.23 (0.92-1.64) | | | .16 |
| CXCL13≥50 | 0.78 (0.70-0.89) | | | | <.0001 | 0.71 (0.44-1.14) | | | .15 |

Abbreviation: SD, standard deviation; BMI, body mass index; ECOG, Eastern Cooperative oncology Group; CEA, Carcinoembryonic antigen; CA 19-9, carbohydrate antigen 19-9; AJCC, American Joint Committee on Cancer; CTx, Chemotherapy; PD-L1, positive programmed death-ligand 1; PD-1, Programmed cell death protein-1.

**Supplementary Table 3** Analysis of clinical factors and immunoprofiles with disease progression in biliary tract cancer

| **Clinical Factor** | **Univariate analysis**  **(n = 54, event = 15)** | | | | **Multivariate analysis**  **(n = 37, event = 10)** | | | |
| --- | --- | --- | --- | --- | --- | --- | --- | --- |
|  | **HR (95% CI)** | | | ***P*-value** | **HR (95% CI)** | | | ***P*-value** |
| **Age (years)** | 0.99 (0.97-1.02) | | | .76 |  |  |  |  |
| **Sex** |  |  |  |  |  |  |  |  |
| Female | ref | | |  |  |  |  |  |
| Male | 1.09 (0.85-1.41) | | | .47 |  |  |  |  |
| **BMI** | 1.00 (0.96-1.04) | | | .91 |  |  |  |  |
| **ECOG** |  |  |  |  |  |  |  |  |
| 0 | ref | | |  |  |  |  |  |
| 1 | - | | |  |  |  |  |  |
| 2 | 0.75 (0.67-0.85) | | | <.0001 |  |  |  |  |
| **Smoking** |  |  |  | .21 |  |  |  |  |
| Never | ref | | |  |  |  |  |  |
| Former | 0.82 (0.65-1.05) | | | .11 |  |  |  |  |
| Current | 1.03 (0.70-1.51) | | | .88 |  |  |  |  |
| **CEA (n = 40)** | 0.99 (0.99-1.00) | | | .018 | 0.99 (0.99-1.00) | | | .025 |
| **CA 19-9 (n = 53)** | 1 | | | .095 |  |  |  |  |
| **Operation after CTx** |  |  |  |  |  |  |  |  |
| NO | ref | | |  | ref | | |  |
| YES | 1.66 (1.07-2.59) | | | .023 | 2.05 (1.71-2.45) | | | <.0001 |
| **Mass size** | 0.99 (0.97-1.02) | | | .66 |  |  |  |  |
| **Stage** |  |  |  | .80 |  |  |  |  |
| II | ref | | |  |  |  |  |  |
| III | 0.86 (0.54-1.36) | | | .52 |  |  |  |  |
| IV | 0.89 (0.56-1.43) | | | .63 |  |  |  |  |
| **Metastasis** |  |  |  | .76 |  |  |  |  |
| None | ref | | |  |  |  |  |  |
| Liver | 0.92 (0.69-1.25) | | | .61 |  |  |  |  |
| Other organs | 1.13 (0.80-1.60) | | | .48 |  |  |  |  |
| Both | 0.93 (0.63-1.37) | | | .70 |  |  |  |  |
| **IHC marker** | **Univariate analysis**  **HR (95% CI)** | |  | ***P*-value** | **Multivariate analysis**  **HR (95% CI)** | |  | ***P*-value** |
| **PD-L1 (n = 51)** |  |  |  |  |  |  |  |  |
| PD-L1<1% | ref | | |  |  |  |  |  |
| 1%≤PD-L1<50% | 0.83 (0.63-1.13) | | | .24 |  |  |  |  |
| PD-L1≥50% | - |  |  |  |  |  |  |  |
| **PD-1 (n = 38)** |  |  |  | .83 |  |  |  |  |
| PD-1<2 | ref | | |  |  |  |  |  |
| 2≤PD-2<20 | 0.94 (0.69-1.27) | | | .68 |  |  |  |  |
| PD-1≥20 | 1.05 (0.67-1.64) | | | .83 |  |  |  |  |
| **FOXP3 (n = 38)** |  |  |  | .98 |  |  |  |  |
| FOXP3<4 | ref | | |  |  |  |  |  |
| 4≤FOXP3<30 | 1.03 (0.74-1.43) | | | .87 |  |  |  |  |
| FOXP3≥30 | 1.01 (0.67-1.54) | | | .95 |  |  |  |  |
| **CD8 (n = 38)** |  |  |  | .73 |  |  |  |  |
| CD8<100 | ref | | |  |  |  |  |  |
| 100≤CD8<200 | 1.18 (0.75-1.86) | | | .47 |  |  |  |  |
| CD8≥200 | 1.10 (0.64-1.91) | | | .72 |  |  |  |  |
| **CXCL13** |  | | |  |  |  |  |  |
| CXCL13<100 |  |  |  | NA |  |  |  |  |
| 100≤CXCL13<200 |  |  |  | NA |  |  |  |  |
| CXCL13≥200 |  |  |  | NA |  |  |  |  |
| **CXCL13T (n = 48)** |  |  |  | <.0001 |  |  |  | .003 |
| CXCL13<1 | ref | | |  | ref | | |  |
| 1≤CXCL13<50 | 0.84 (0.57-1.24) | | | .38 | 1.05 (0.59-1.85) | | | .86 |
| CXCL13≥50 | 0.69 (0.59-0.81) | | | <.0001 | 0.76 (0.63-0.90) | | | .001 |

Abbreviation: SD, standard deviation; BMI, body mass index; ECOG, Eastern Cooperative oncology Group; CEA, Carcinoembryonic antigen; CA 19-9, carbohydrate antigen 19-9; AJCC, American Joint Committee on Cancer; CTx, Chemotherapy; PD-L1, positive programmed death-ligand 1; PD-1, Programmed cell death protein-1.
